# Ordered nanopore arrays with large interpore distances *via* one-step anodization†
†Electronic supplementary information (ESI) available. See DOI: 10.1039/c8nr02215a


**DOI:** 10.1039/c8nr02215a

**Published:** 2018-04-16

**Authors:** I. Mínguez-Bacho, F. Scheler, P. Büttner, K. Bley, N. Vogel, J. Bachmann

**Affiliations:** a Department of Chemistry and Pharmacy , Friedrich-Alexander University of Erlangen-Nürnberg , Egerlandstr. 1 , 91058 Erlangen , Germany . Email: julien.bachmann@fau.de; b Institute of Particle Technology , Friedrich-Alexander University of Erlangen-Nürnberg , Haberstr. 9a , 91058 Erlangen , Germany . Email: nicolas.vogel@fau.de; c Institute of Chemistry , Saint Petersburg State University , 26 Universitetskii Prospect , Saint Petersburg , Petergof 198504 , Russia

## Abstract

Preparation of pre-patterned alumina substrates using bottom-up techniques compatible with nanotechnology applications is still a challenge. We present a novel methodology to achieve superior order in ‘anodic’ alumina with large interpore distances by a convenient one-step anodization process. The use of transparent insulators renders such anodic layers applicable as templates for nanostructured photovoltaic or photoelectrochemical devices.

## Abstract 2

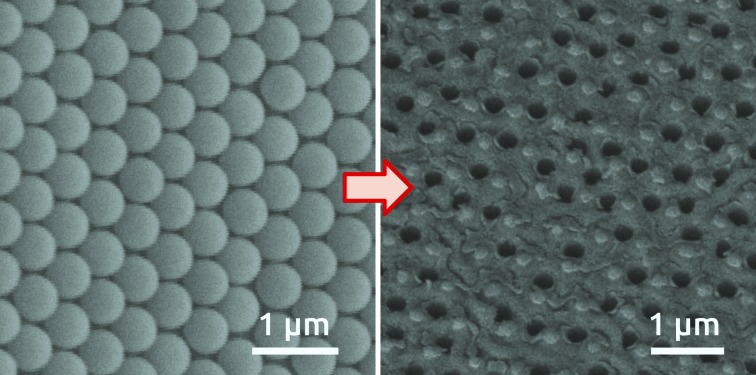
We prepare arrays of cylindrical pores featuring large periods (460 nm and 600 nm) by anodization of aluminum. A self-ordered monolayer of nanospheres drives the subsequent pore ordering and yields a quality of order significantly improved with respect to the traditional two-step anodization procedure.

## 

Porous anodic aluminum oxide (AAO) is a popular template system that provides self-ordered arrays of parallel, straight, cylindrical pores of tunable lengths and diameters.^1^ It has been exploited towards a variety of applications such as sensing, filtration, electrochemistry, batteries or photovoltaics.^2^ Interpore distances (*D*_int_) obtained under the self-ordering conditions associated with conventional mild anodization (MA) of aluminum for sulfuric, oxalic and phosphoric acid based electrolytes are limited to ∼65, ∼105, and ∼450 nm, respectively.^3^ Hard anodization (HA) has allowed for a broadening of the self-ordering regimes to a wider and almost continuous range of *D*_int_ from 70 to 380 nm in electrolytes based on sulfuric, oxalic, and phosphoric acids and their mixtures.^4^ Nevertheless, both MA and HA require a two-step anodization process to achieve parallel arrays of straight nanopores under the self-ordering regime. Additionally, the size of perfectly ordered domains is limited to a few square micrometers. This becomes more evident in AAO films with larger interpore distances such as those based on phosphoric acid electrolytes, where the domains with self-ordering contain a low number of pores. Furthermore, obtaining a *D*_int_ beyond 450–500 nm with self-ordered nanopores presents difficulties due to the necessity of applying high voltages which may lead to breakdown effects. Organic acids (also in combination with others such as oxalic and phosphoric acids) can be used under such large voltages, although the quality of self-ordering is reduced in comparison with conventional anodization.^5^ Recently, important findings have been made towards the achievement of new self-ordering regimes by using electrolytes based on etidronic acid (*D*_int_: 400–670 nm at 165–280 V) and citric acid electrolytes (*D*_int_: 645–884 nm at 350–390 V).^6^ These high voltages also result in high growth rates which make the AAO film thickness difficult to control and require several hours to achieve self-ordering. In short, an experimentally simple process is still missing for the reliable production of such large pore distances (a) in a single anodization step, (b) with a high degree of order obtained exclusively with bottom-up techniques, and (c) in the absence of any opaque (metallic) layer. Such a process would significantly extend the application scope of these important nanoscale architectures.

While some nanotechnology applications are compatible with pore lengths on the order of tens of microns, others rely on much shorter pores, 5 μm and below.^7^ Long pores, and correspondingly thick porous layers, are ideally produced from a bulk aluminum sheet *via* the traditional “two-step” anodization procedure. Their thin counterparts, however, cannot be handled as free-standing membranes, and require an underlying substrate for mechanical stability. Since the substrate is often different from metallic aluminum, thin aluminum films evaporated on an appropriate substrate are the logical solution to obtain AAO architectures with comparably short pores.^8^ However, such typically thin (<1 μm) layers are not amenable to two-step anodization, which consumes a large thickness of aluminum. These limitations therefore call for alternative methods designed to enforce appropriate order at the very beginning of anodization, and thereby circumvent the need for the two-step procedure. This is possible by using pre-patterned substrates. A most parallel technique to create long range ordered patterns is imprint of nanoindentations *via* stamps.^9^ However, this method requires substrates which are able to withstand high mechanical pressures to replicate the pattern of the stamp and requires nanofabrication tools for their fabrication. Therefore, alternative techniques, providing simple, inexpensive and reliable access for pre-patterning of thin or fragile Al substrates are necessary. Methods such as focused ion beam or nanoindentation *via* the tip of a scanning microprobe are suitable,^10^ but require sophisticated equipment, are time consuming due to their serial nature and are limited to small areas.

A technique that yields perfectly ordered structures over large areas is laser interference lithography (LIL).^11^ This technique, however, requires a dedicated optical setup and is challenging for *D*_int_ < 200 nm. An alternative to LIL is provided by nanosphere lithography (NSL), also called colloidal, or ‘natural’, lithography. This technique employs two-dimensional colloidal monolayers as templates to create surface patterns with high precision and uniformity over large areas.^12^ A colloidal monolayer is preassembled on a substrate and used as a shadow mask for a directed deposition process of materials. After the removal of the colloidal particles, well-defined surface nanostructures with a high degree of order and hexagonal symmetry inherited from the colloidal templates are produced. NSL cannot achieve the perfect order that LIL can produce over several cm^2^ (the ‘crystalline’ domains are typically on the order of tens of microns and can be up to mm sized), but has the advantages of versatility and experimental simplicity. Spherical colloids of silica and polystyrene are commercially available with diameters from below 100 nm up to microns and can also be synthesized using well-established procedures.^13^ Their self-ordering based on Langmuir–Blodgett derived methods only requires basic laboratory glassware and a syringe pump.^14^

In this work, we present a facile and inexpensive method to obtain pores with large *D*_int_ and high degree of order with a large domain size using one-step anodization which also allows for the growth of thin AAO films in a controlled manner. Our method exploits NSL and can be generalized to a variety of planar and hydrophilic substrates. We use a monolayer of spherical polystyrene (PS) colloidal particles (460 or 600 nm in diameter) as a mask and sputter-deposit SiO_2_ onto Al substrates. We chose SiO_2_ because it is chemically inert and has electrically insulating properties, the attributes that are needed to control the start of anodization of the underlying Al foil. An additional benefit is that SiO_2_ exhibits optical transparency, which later allows for applications of the AAO film in photovoltaic or photoelectrochemical devices. This represents an important advantage with respect to the use of metals, which has been reported in related schemes.^15^

In the first step of our procedure, a dispersion of PS spheres is added onto the air–water interface under conditions appropriate to form a hexagonally close-packed monolayer. Colloids are synthesized by surfactant-free emulsion polymerization with acrylic acid as a co-monomer as described elsewhere.13*a* Self-assembly is performed using the air–water interface as a template following a protocol from the literature for PS spheres^14^ (see the Experimental section). The high degree of order of the colloidal monolayer at the air–water interface is evidenced by the bright colors arising from diffraction grating effects (Fig. S1 in the ESI†). The pre-assembled monolayer is then transferred to an electropolished aluminum substrate (Fig. 1), without compromising the order, as evidenced from the diffraction grating colors. The pre-pattering process involves four steps schematically represented in Fig. 1d and explained in detail in the Experimental section. Scanning electron microscopy (SEM) images of the PS spheres deposited on the aluminum substrate are shown in Fig. 1e. The high quality of order, with single crystalline, hexagonal closed-packed domains spanning hundreds of square micrometers, can be observed in the SEM micrographs (Fig. S2†). The PS spheres are then plasma-etched to reduce their size, resulting in a non-close-packed arrangement. The gaps created around the PS spheres, resulting from a shrinkage of about 15% of the initial diameter, are clearly visible in Fig. 1f. The shrinkage of PS spheres is necessary to avoid their coalescence during the subsequent thermal treatment and to open space for the patterning of the substrate. The thermal annealing step serves to increase the contact area between the colloidal particles and the Al substrate, as a way of correcting for the imperfect directionality of deposition in the next step. A SiO_2_ film of 50 nm thickness is subsequently sputter-coated onto the substrate with the colloidal monolayer template (Fig. 1g), after which the PS spheres are detached from the substrate. As a result, a hexagonal close-packed array of openings in the SiO_2_ layer is created, in which the underlying Al is exposed (Fig. 1h). These openings reproduce the hexagonal arrangement of the PS spheres, and each is surrounded by ridges corresponding to the gaps between the spheres.

**Fig. 1:**
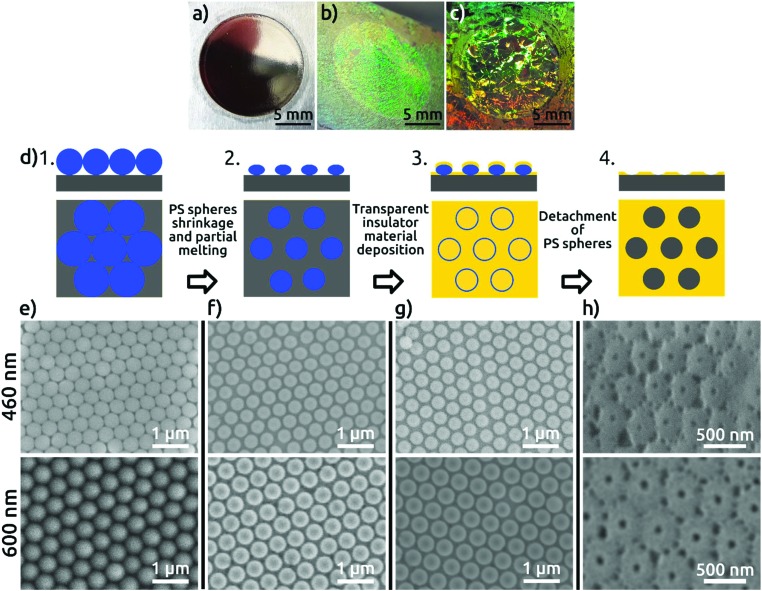
Photographs of (a) bare Al foil with an electropolished circular area of 1.4 cm diameter, (b) with a monolayer of PS spheres of 460 nm and (c) 600 nm diameter transferred to the aluminum surface; (d) scheme of the four fabrication steps of the SiO_2_ patterned Al foil. SEM micrographs of the respective fabrication steps for 460 nm (upper micrographs) and 600 nm (lower micrographs); (e) as-deposited PS spheres, (f) shrunk PS spheres with diameter of 400 nm (upper micrographs) and 520 nm (lower micrographs), (g) after deposition of 50 nm of SiO_2_ by radio-frequency (RF) sputtering, (h) hexagonally patterned SiO_2_ layer with distances of 460 nm and 600 nm on Al foil after detaching the PS spheres (viewed at an angle of 40°).

The Al foil hexagonally pre-patterned with the colloidal spheres is then anodized at 0 °C in phosphoric acid under 195 V for 30 minutes. For comparison, two reference samples are also anodized under the same conditions: an electropolished Al foil without any pre-patterning, and an Al foil submitted to a preliminary first anodization for 24 hours, following the traditional strategy to increase pore ordering.^1^,^3^ Top-view SEM micrographs of the surface of the resulting AAO films and their respective self-correlation images (SCIs)^16^ evidence marked differences among these three different samples (Fig. 2). The detailed analysis of the ordering of nanopores based on the SCIs obtained from the SEM micrographs in Fig. 2 highlights the superior order in the sample fabricated by the colloidal templating method. The complete absence of order in AAO nanostructures after first anodization (Fig. 2a), is evidenced by a uniform ring, corresponding to the first-neighbor *D*_int_ = 420 nm (Fig. 2b), without showing any signs of periodic ordering. The AAO film obtained after a ‘traditional’ second anodization is presented in Fig. 2c, which evidences local self-ordering. In the corresponding SCI (Fig. 2d), the central spot is now complemented by two concentric rings of approximately hexagonal shapes. This demonstrates that order reaches at least the second neighbors with a regular *D*_int_ = 470 nm. Fig. 2e, however, which shows the sample anodized once, but with the NSL pre-patterning, features a hexagonal order of much superior quality. The corresponding SCI exhibits an almost perfect extended hexagonal structure evidencing the long range of nanopore order, with *D*_int_ = 470 nm (Fig. 2f). Fig. 3S† shows the lateral size of the hexagonally ordered domains to be several tens of micrometers.

**Fig. 2:**
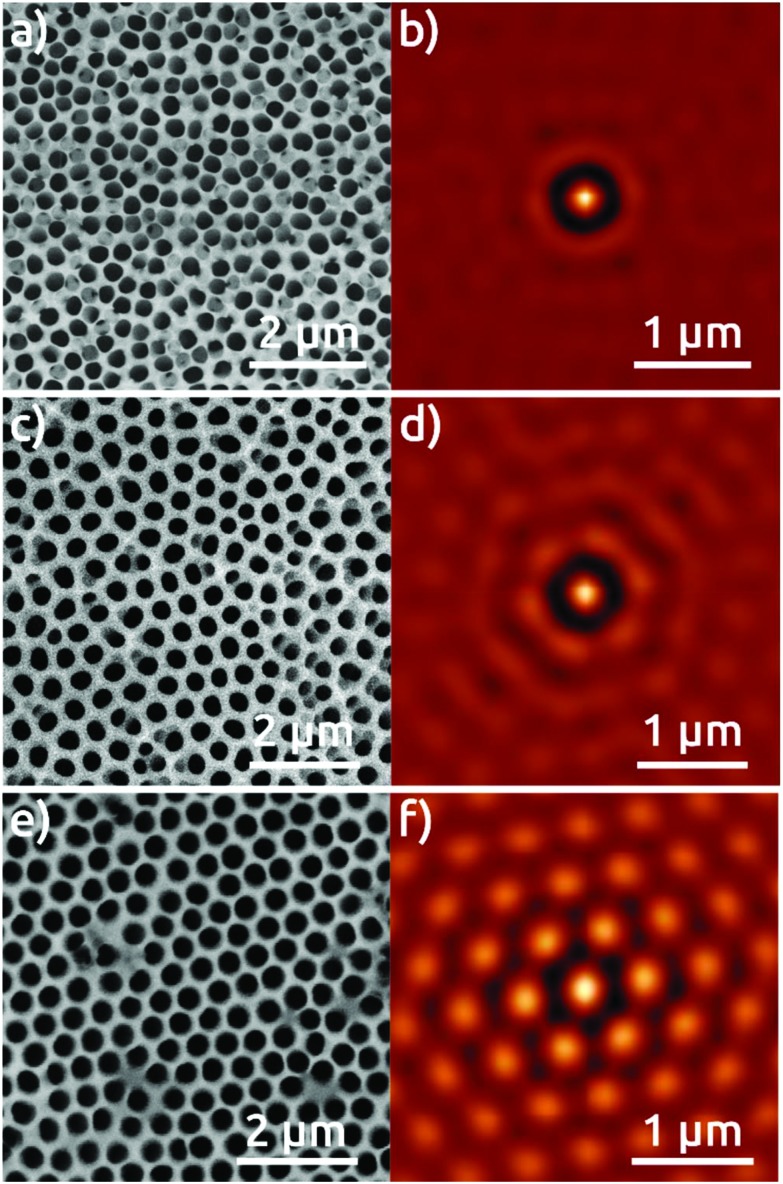
Top view SEM micrographs of the AAO film grown in a 0.1 M phosphoric acid electrolyte applying 195 V after: (a) first anodization of electropolished Al foil, (c) second anodization, (e) anodization of Al foil with pre-patterned structures, and (b, d, and f) their respective self-correlation images (SCIs).

This extremely high degree of order is also obtained with the 600 nm sphere system, although some limitations remain. Fig. 3 shows in top view the AAO film obtained upon anodization in citric acid for first anodization (Fig. 3a), second anodization (Fig. 3c), and for the single anodization on Al pre-patterned with the 600 nm spheres (Fig. 3e). The SCI of the micrograph after first anodization (Fig. 3b) presents only one ring corresponding to the fixed distance to the nearest neighbors, and no longer-scale order. The SCI after second anodization shows a localized improvement in the short range (Fig. 3d), where the central spot is surrounded by a hexagonal ring. Fig. 3e and f show an evidently improved long-range hexagonal order with the pre-patterned Al surface after single anodization. The SCI reveals a perfectly hexagonal arrangement of spots around the central one which retain their individuality over microns. These characteristics are reproduced over large areas of hundreds of μm^2^ (Fig. S4†), although some sites do not seem to nucleate any pores at all. This NSL approach does yield a significant improvement of order with respect to anodization in the absence of nanospheres. Correspondingly, our NSL approach affords access to a range of periods that are not achievable otherwise, and which have not been demonstrated in NSL-based work so far.

**Fig. 3:**
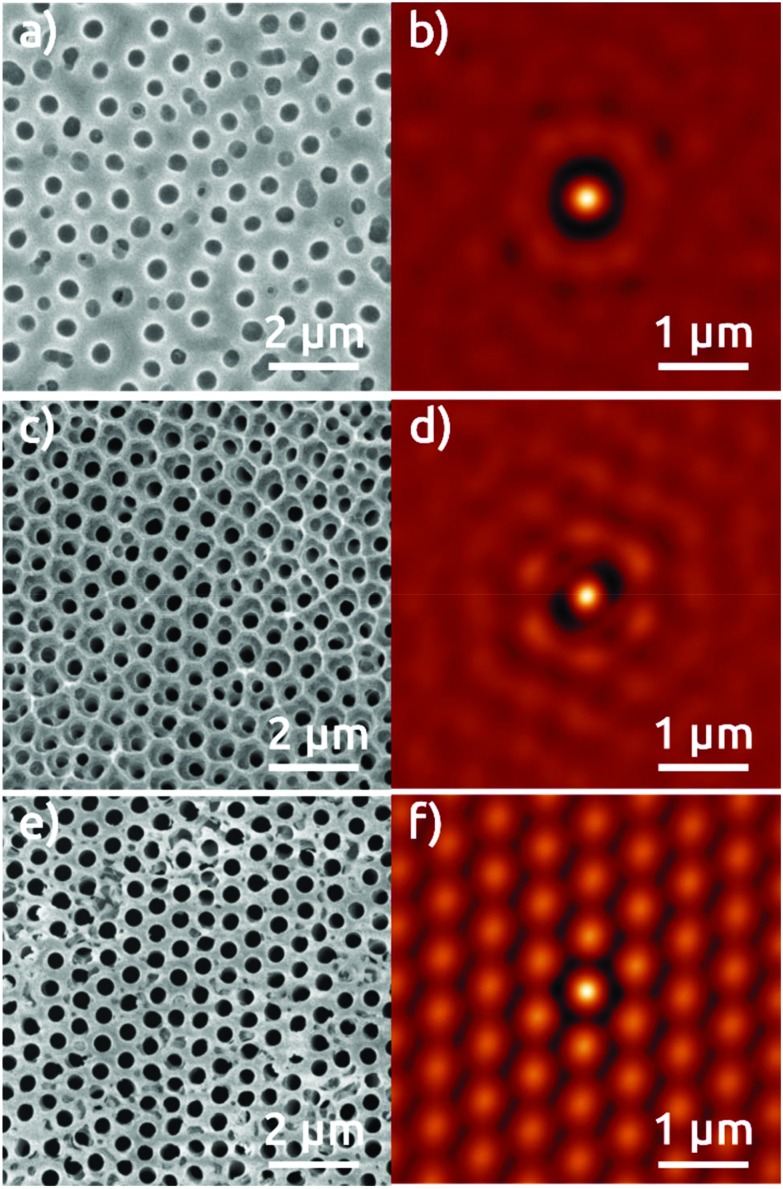
Top view SEM micrographs of the AAO film grown in a 1.5 M citric acid electrolyte applying 270 V after: (a) first anodization of electropolished Al foil, (c) second anodization, (e) anodization of Al foil with pre-patterned structures, and (b, d, and f) their respective SCIs.

A quantitative comparison of the order quality is presented with normalized ordering factors, calculated (as defined in the literature)^16^ from the SCIs of micrographs with similar areas and numbers of pores, in Fig. 4. The normalized ordering factor obtained for the AAO films grown in phosphoric acid based electrolytes increased significantly from 0.21 to 0.30 by use of the popular “two-step” anodization procedure, but even more impressively to 0.60 for our pre-patterned AAO nanopore arrays. Correspondingly, the normalized ordering factor obtained for anodization under citric acid electrolyte conditions presents values of 0.14 and 0.27 for first and second anodization, respectively. This value increases up to 0.43 for the NSL patterned AAO film. Independent of the period, the SiO_2_ layer detaches from the surface upon ‘pore widening’ of AAO, which partially dissolves the aluminum oxide by immersion in a 10 wt% phosphoric acid solution (Fig. S5†).

**Fig. 4:**
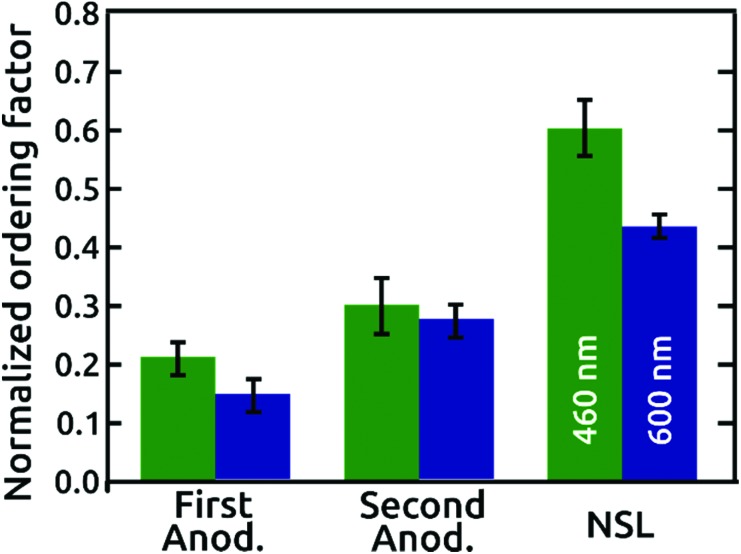
Normalized ordering factors of the AAO nanopore arrays extracted from the respective SCIs of the AAO films grown in phosphoric (green) and citric acid (blue) based electrolytes. Normalized ordering factors are shown for first and second anodization, and first anodization on NSL patterned surfaces with 460 and 600 nm periods, respectively.

The high degree of order observed at the pore openings should be replicated at the other pore extremity, that is, on the Al substrate underneath the AAO film. This aspect has mostly been overviewed in previous reports of NSL combined with anodization.12b,^15^,^17^ A cross-sectional view of our 460 nm period pre-patterned AAO film, Fig. S6a,† substantiates that the pores have grown perpendicular to the surface and parallel to each other. We note that the AAO material presents gaps between second-nearest neighbors, with a distance between pores of about 815 nm. Applying basic trigonometry from this value, we calculate a nearest-neighbor interpore distance of 470 nm, which fits exactly with the measurement taken from the SCI in Fig. 2f. For further confirmation, we dissolve the pre-patterned AAO film to observe the pattern left on the Al substrate. The micrograph shows a highly ordered pattern of dimples (Fig. 5a). The analysis of the corresponding SCI reveals an almost perfect hexagonal distribution of individual spots indicating a highly reproducible pattern over a large area with a period of 485 nm (Fig. 5b). These patterns are reproduced over large areas greater than 900 μm^2^ (Fig. S7†).

**Fig. 5:**
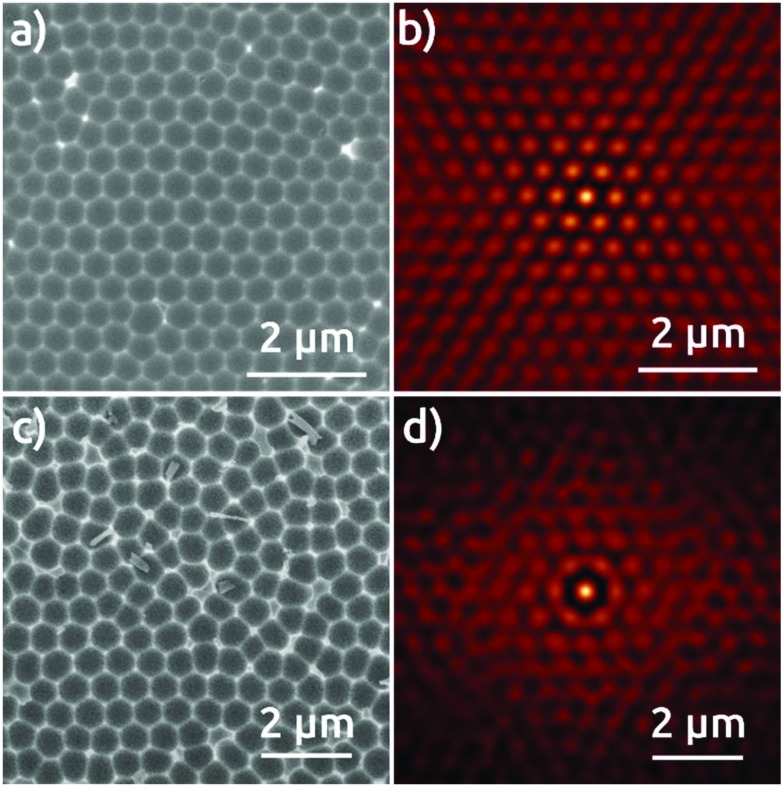
Top view SEM micrographs of the Al substrate surface after dissolving the AAO film grown on pre-patterned structures of (a) 460 nm and (c) 600 nm and (b, d) the respective SCIs.

However, anodization in organic acids producing porous AAO with interpore distances around *D*_int_ = 600 nm does not belong to a well-established self-ordering regime of anodization. The cross-sectional view of the 600 nm period pre-patterned alumina substrates reveals pores that mainly do not grow perfectly parallel to each other (Fig. S6b†). The micrograph of the exposed Al substrate after dissolving the pre-patterned AAO film reveals ordered domains of a few micrometers only (Fig. 5c). This is reflected in the SCI (Fig. 5d). However, our procedure enables one, for the first time, to create self-ordered domains of pores with a period beyond 500 nm with a single anodization step (Fig. S8†).

## Conclusions

Taken together, these results demonstrate the feasibility of a NSL method for the creation of nanoporous AAO films with large interpore distances. A transparent insulator material (SiO_2_) is used as a sputtered mask to guide the initiation of pore formation. This one-step anodization process reduces the fabrication times of self-ordered structures substantially and significantly increases the areas of perfectly ordered nanopore domains. The resulting AAO films are transparent without any further treatments. They can be applied directly as templates for the fabrication of highly ordered nanostructured photoactive composites, and towards the systematic study of interface *versus* transport phenomena in applications such as photovoltaics and photoelectrochemistry.

## Conflicts of interest

There are no conflicts to declare.

## Supplementary Material

Supplementary information
