# Supplementary material for: Ordered nanopore arrays with large interpore distances via one-step anodization
Source: Nanoscale. 2018 Apr 16;10(18):8385–90. doi: 10.1039/c8nr02215a (PMC5944388; doi:10.1039/c8nr02215a)
Supplement: Supplementary file 1 [file NR-010-C8NR02215A-s001.pdf]

Electronic Supplementary Material (ESI) for Nanoscale.
This journal is © The Royal Society of Chemistry 2018

Electronic Supplementary Information to

Ordered Nanopore Arrays with Large Interpore Distance via One-Step Anodization

Ignacio Mínguez-Bacho, Florian Scheler, Pascal Büttner, Karina Bley,

Nicolas Vogel and Julien Bachmann**

Dr. I. Mínguez-Bacho, F. Scheler, P. Büttner, Prof. J. Bachmann.

Department of Chemistry and Pharmacy, Friedrich-Alexander University of Erlangen-Nürnberg, Egerlandstr. 1, 91058 Erlangen, Germany.

E-mail: julien.bachmann@fau.de

Dr. K. Bley, Prof. N. Vogel.

Institute of Particle Technology, Friedrich-Alexander University of Erlangen-Nürnberg, Haberstr. 9a, 91058 Erlangen, Germany.

E-mail: nicolas.vogel@fau.de

Prof. J. Bachmann.

Institute of Chemistry, Saint Petersburg State University, 26 Universitetskii Prospect, Saint Petersburg, Petergof 198504, Russia.

Experimental Section

Colloidal polystyrene spheres self-assembly: Colloids are synthesized by surfactant-free emulsion polymerization with acrylic acid as co-monomer as described elsewhere.[1] Self-assembly is performed using the air-water interface as a template following a protocol from literature for PS spheres.[2] A colloidal dispersion of polystyrene (PS) spheres (5 wt%) of 460 nm diameter is diluted with ethanol (25 vol%) to facilitate spreading on the air/water surface and HCl 0.1 M (25 vol%) to enable floating of the spheres on the interface, resulting in a final dispersion of PS spheres of 2.5 vol%. Another colloidal solution of PS spheres with diameters of 600 nm (8 wt%) is diluted in ethanol (50 vol%), resulting in a final dispersion of PS spheres of 4 wt%. These respective dispersions are introduced in a syringe and added to the air-water interface via a tilted glass slide forming 45 ° angle to the water surface at a pumping rate of 40 µl min^{-1} for the dispersion of 460-nm spheres. In case of the dispersion of 600 nm spheres the needle is placed at 90 ° angle introducing partially the tip to the surface of the water and pumping the dispersion to the air-water interface at a rate of 70 µl min^{-1}. The colloids are added to the air-water interface until the whole surface is covered by a closed-packed monolayer (Figure S1).

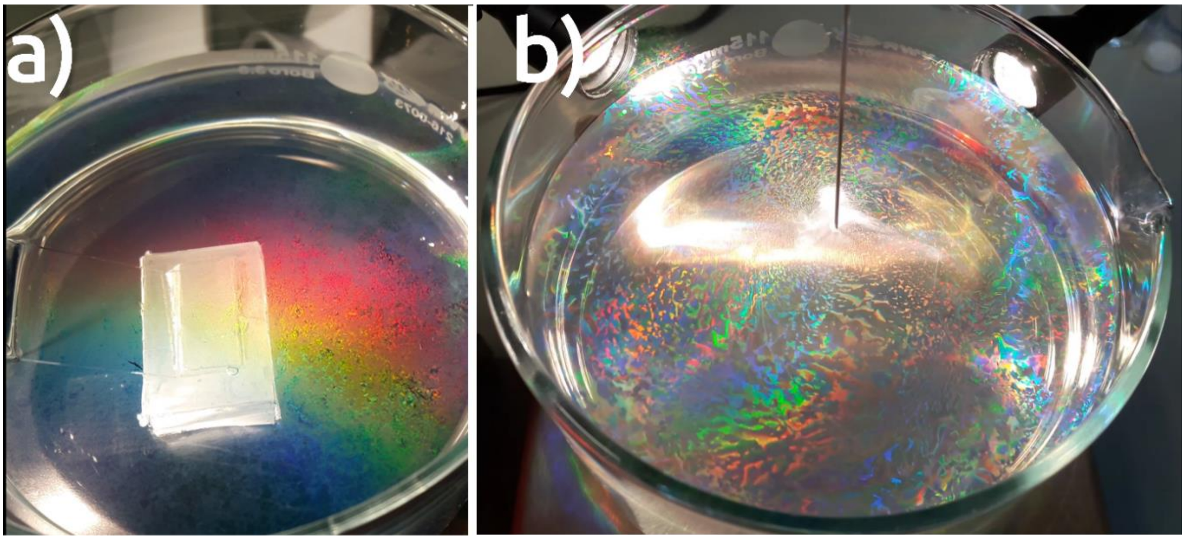

Figure S1. Photographs of PS spheres closed-packed monolayer added onto the air-water interface via a tilted glass slide. The size of the PS spheres is a) 460 nm and b) 600 nm.

Transfer of the pre-assembled colloidal monolayer is achieved by manually immersing the substrate into the water subphase and removal under a shallow angle to deposit the interfacial colloidal monolayer. After drying, a colloidal monolayer with high order is deposited on the substrate.

Preparation of Al substrates: Al foil (99.999% purity) substrates are electropolished in a mixture of perchloric acid (25 vol%) and ethanol (75 vol%) electrolyte at 5 °C under vigorous stirring and an applied voltage of 20 V during 5 minutes. These electropolished Al foil substrates are subjected to plasma treatment (Ar gas flow rate of 5 sccm; power: 100 W; time: 5 minutes) to render the surface more hydrophilic immediately before transferring the PS spheres onto the substrate.

Pre-patterning of Al substrates: A schematic illustration of the fabrication step of the pre-patterned Al substrates is presented in Figure 2a. First, electropolished Al substrates are vertically immersed through the PS spheres monolayer at the air-water interface, lifted manually at an angle of 45 degrees and left to dry at a similar angle. Second, the PS spheres monolayer on the electropolished Al foil substrates with are etched under plasma treatment (Ar gas flow rate: 5 sccm; power: 50 W; time: 5.5 minutes) to shrink the PS spheres. Third, the PS spheres are partially melted in an oven (95 °C, 1 minute) to increase the contact area with the Al surface. 50 nm thick SiO_2 layer is deposited on the PS spheres/Al substrate by radio frequency (RF) sputtering on a rotating substrate at 0.3 Å s^{-1} (power: 50 W; Ar gas flow: 5 sccm, chamber pressure: $3.2 \cdot 10^{-3}$ Torr; model CRC 622 by Torr International, Inc.). Fourth, the PS spheres are detached by immersing the sample in toluene under sonication for one minute and then, rinsed in acetone, isopropanol and water.

Anodization of Al substrates: bare electropolished and pre-patterned Al foil substrates are placed in an electrochemical cell with a cooling system set at a nominal temperature of 0 °C. The resulting AAO film obtained after first anodization on bare Al substrates is dissolved in

chromic acid solution (1.8 wt% CrO_3, 6 wt% H_3PO_4) and then anodized again under the same experimental conditions. After anodization, the resulting samples are immersed in diluted phosphoric acid solution 10 wt% concentration at 45 °C for 40 minutes to widen the nanopores.

a) Phosphoric acid-based electrolyte: Bare and pre-patterned Al substrates based on PS spheres of 460 nm are anodized in phosphoric acid-based electrolyte with an initial concentration of 0.05 M and increased to 0.1 M after 10 minutes of anodization at 195V with overhead mechanical stirring. Bare Al foil substrates are anodized during 24 hours for the first anodization. The second anodization is carried out for one hour. The total anodization time on pre-patterned Al substrates is 30 minutes.
b) Citric acid-based electrolyte: Bare and pre-patterned substrates based on PS spheres of 600 nm are anodized in citric acid-based electrolyte with a concentration of 1.5 M under 270 V with overhead mechanical stirring. Bare Al foil substrates are anodized during 1 hour for the first anodization. The second anodization is carried out for 10 minutes. The anodization time on pre-patterned Al substrates is 10 minutes. Prior to the anodization on pre-patterned substrates the sample is immersed in chromic acid solution during 20 min at room temperature to thin down the native oxide layer and facilitate the anodization.

Characterization and analysis: Scanning electron micrographs are taken on a Jeol JSM 6400 equipped with energy-dispersive X-ray spectrometer. Self-correlation image processing is carried out with free software WSxM4.0 develop 12.4.[3]

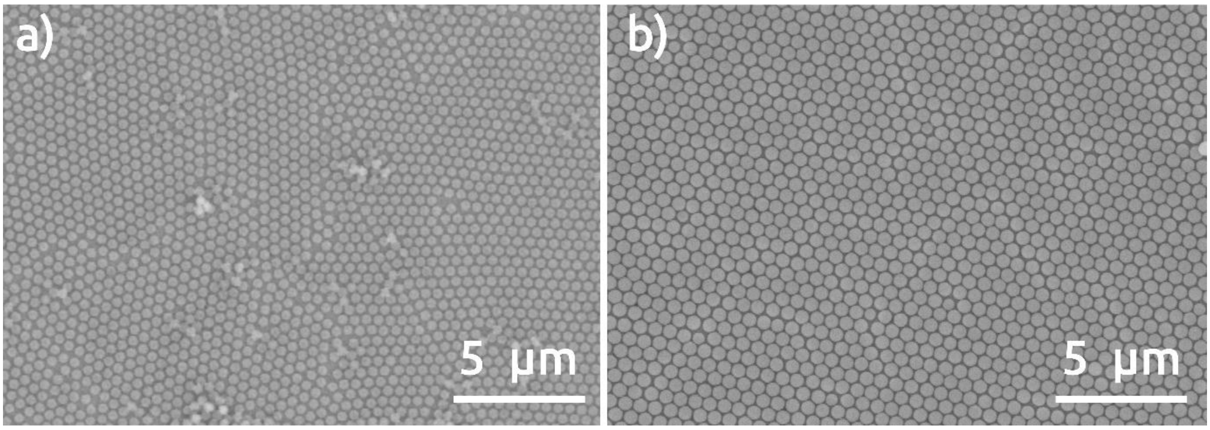

Figure S2. Micrographs of monolayers of shrunk PS spheres with diameters of a) 460 nm and b) 600 nm.

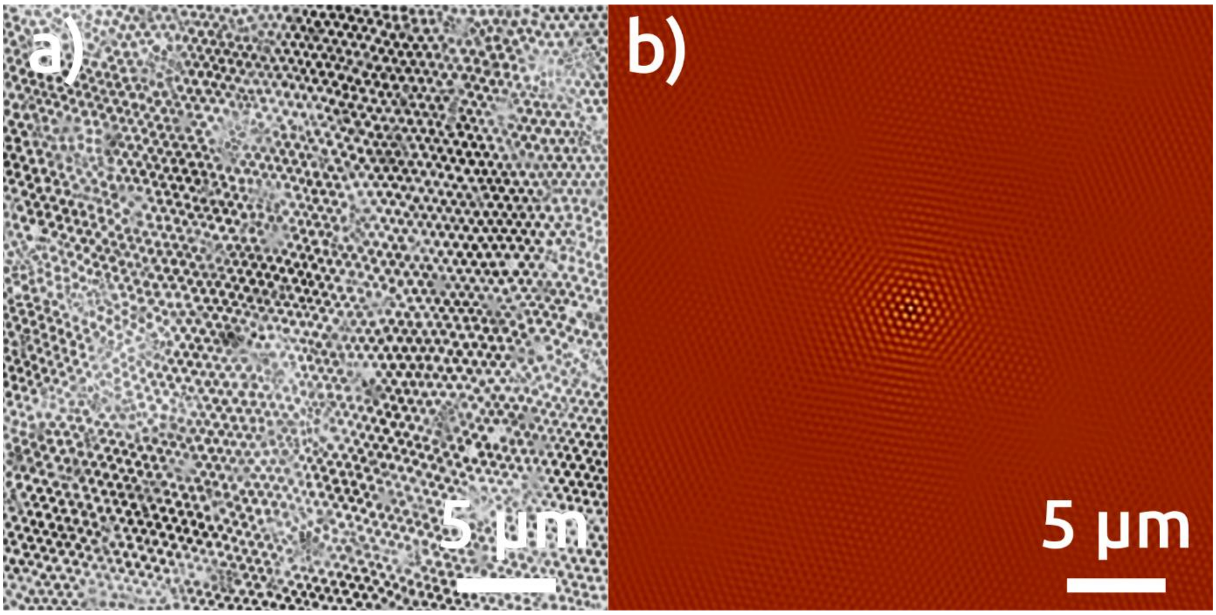

Figure S3. a) Top view SEM micrograph of AAO film surface after anodization of Al foil in 0.1 M phosphoric acid electrolyte under 195 V with pre-patterned structures of 460 nm over an area of 900 μm^2 featuring perfectly ordered domains; b) the corresponding self-correlation image (SCI).

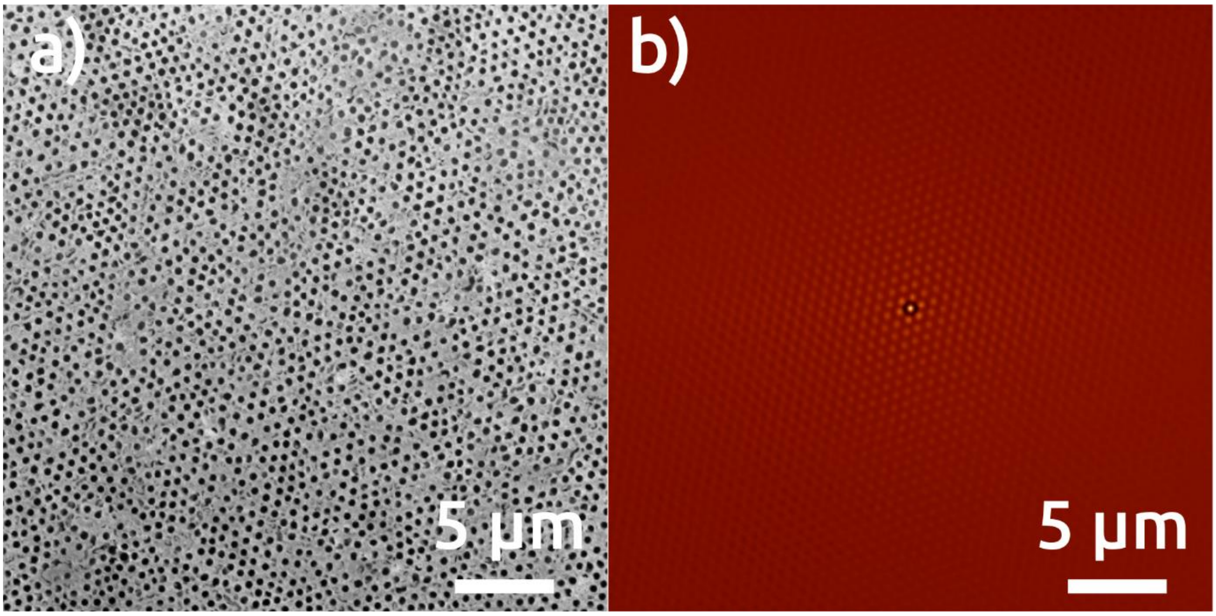

Figure S4. a) Top view SEM micrograph of a) AAO film surface after anodization of Al foil in 1.5 M phosphoric acid electrolyte under 270 V with pre-patterned structures of 600 nm over an area of 900 μm^2 featuring perfectly ordered domains; b) the corresponding SCI.

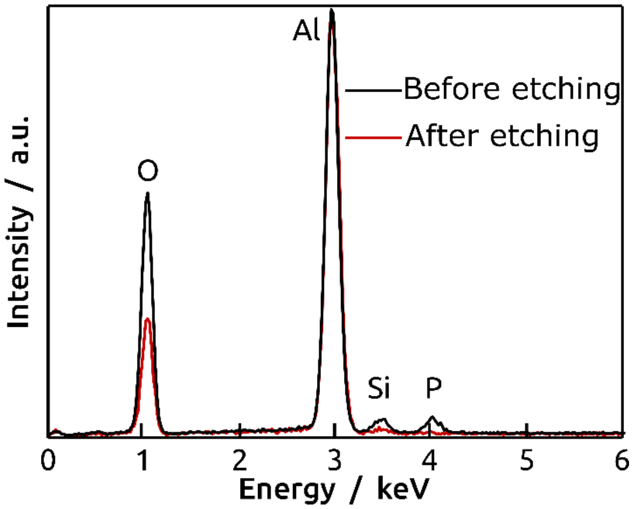

Figure S5. Energy-dispersive X-ray spectra of a pre-patterned AAO film before (black) and after (red) etching with phosphoric acid solution 10 wt% for 40 min at 45 °C. The Si signal after the 'pore widening' is negligible due to SiO_2 removal, as well as the P signal (due to removal of the anion-contaminated outer layer of AAO nanopores).

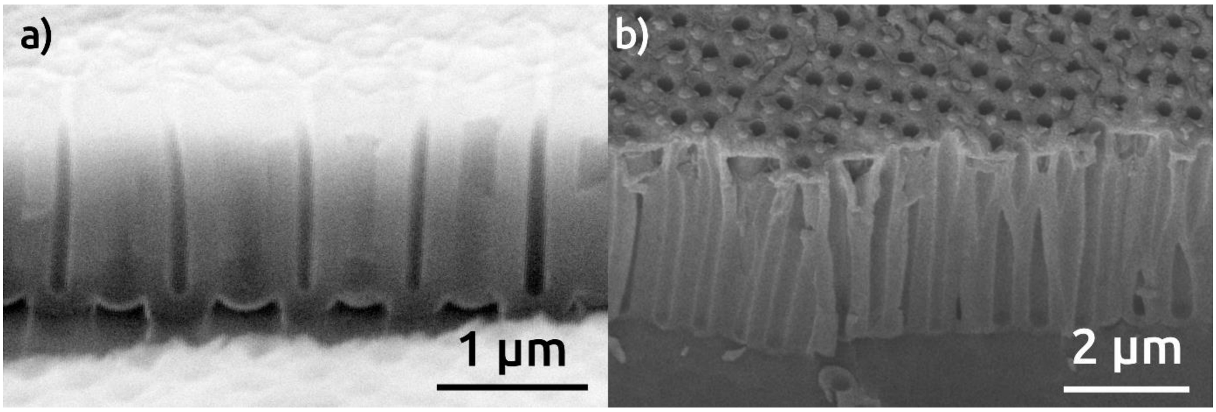

Figure S6. Cross sectional SEM micrographs of AAO film grown on a) 460 nm and b) 600 nm lattice pre-patterned Al substrate.

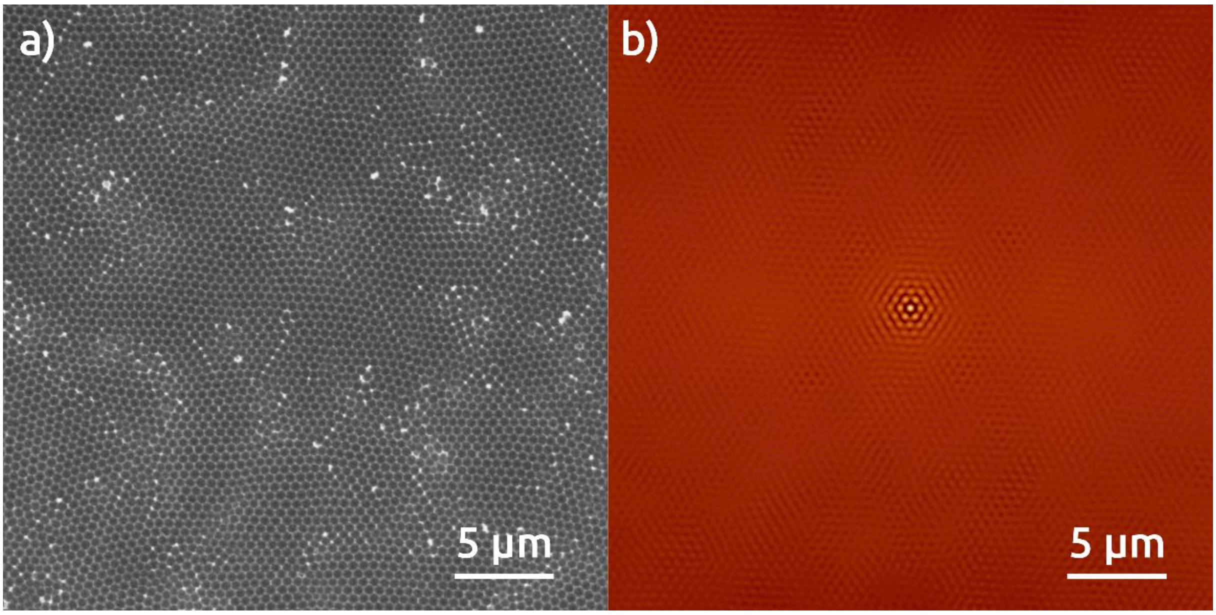

Figure S7. Top view SEM micrograph of a) Al substrate surface after dissolving the AAO film grown on pre-patterned structures of 460 nm over an area of 900 µm^2, b) the corresponding SCI.

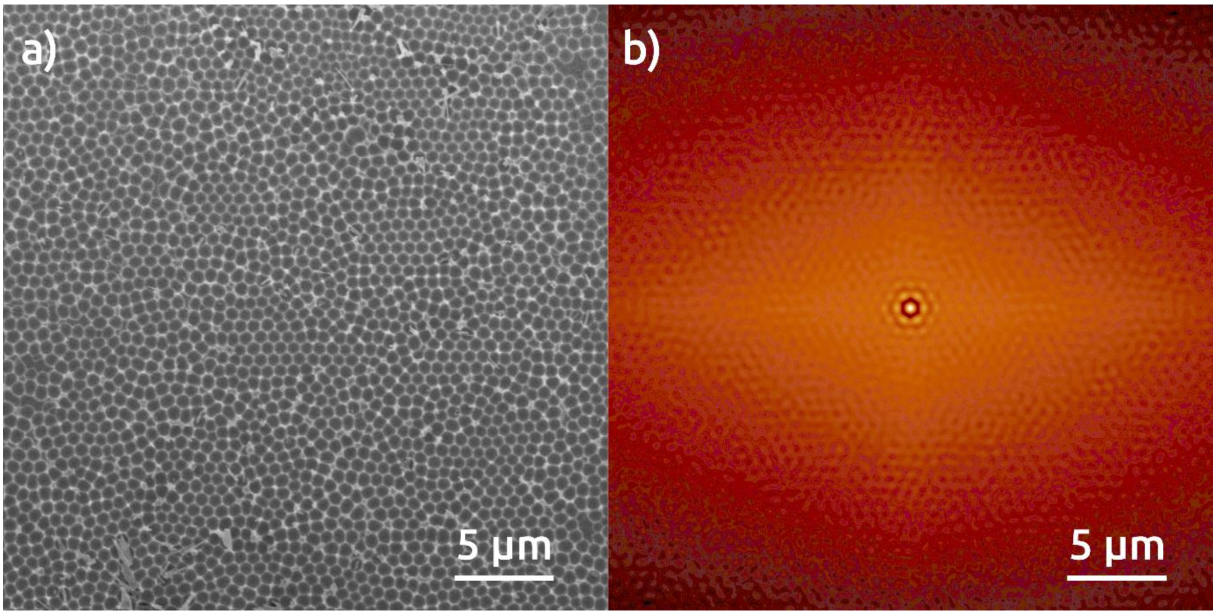

Figure S8. Top view SEM micrograph of a) Al substrate surface after dissolving the AAO film grown on pre-patterned structures of 600 nm over an area of 900 µm^2, b) the corresponding SCI.

1 N. Vogel, L. de Viguerie, U. Jonas, C. K. Weiss, K. Landfester, *Adv. Funct. Mater.,* 2011**, 21**, 3064.
2 N. Vogel, S. Goerres, K. Landfester, C. K. Weiss, *Macromol. Chem. Phys.,* 2011**, 212**, 1719.
3 I. Horcas, R. Fernández, J. M. Gómez-Rodriguez, J. Colchero, J. Gómez-Herrero, A. M. Baro, *Rev. Sci. Instrum.,* 2007, **78**, 013705.
